# An Increase in the Levels of Middle Surface Antigen Characterizes Patients Developing HBV-Driven Liver Cancer Despite Prolonged Virological Suppression

**DOI:** 10.3390/microorganisms9040752

**Published:** 2021-04-02

**Authors:** Giuseppina Brancaccio, Romina Salpini, Lorenzo Piermatteo, Matteo Surdo, Vanessa Fini, Luna Colagrossi, Marco Cantone, Arianna Battisti, Yasunori Oda, Domenico Di Carlo, Francesca Ceccherini-Silberstein, Carlo Federico Perno, Giovanni Battista Gaeta, Valentina Svicher

**Affiliations:** 1 Infectious Diseases, University Hospital of Padua, 35128 Padua, Italy; ggbrancaccio@gmail.com; 2 Department of Experimental Medicine, Tor Vergata University, 00133 Rome, Italy; rsalpini@gmail.com (R.S.); lorenzo.piermatteo@uniroma2.it (L.P.); battisti.arianna@gmail.com (A.B.); ceccherini@med.uniroma2.it (F.C.-S.); 3 Molecular Genetics Laboratory, Eurofins Genoma, 00138 Rome, Italy; surdo@laboratoriogenoma.it (M.S.); fini@laboratoriogenoma.it (V.F.); 4 Department of Diagnostic Medicine and Laboratory, Bambin Gesù Children’s Hospital, 00165 Rome, Italy; luna_colagrossi@yahoo.it (L.C.); perno@uniroma2.it (C.F.P.); 5 Department of Mental Health and Public Medicine, Section of Infectious Diseases, University of Campania “Luigi Vanvitelli”, 81100 Naples, Italy; marcokant89@gmail.com (M.C.); giovannibattista.gaeta@unicampania.it (G.B.G.); 6 Beacle Inc., Kyoto 600-8305, Japan; y_goh@beacle.com; 7 Paediatric Clinical Research Center “Romeo and Enrica Invernizzi”, University of Milan, 26900 Milan, Italy; pvt.math82@gmail.com

**Keywords:** hepatocellular carcinoma, hepatitis B, HBsAg, HBs isoforms, middle-HBs

## Abstract

Hepatitis B virus (HBV) contains three surface glycoproteins—Large-HBs (L-HBs), Middle-HBs (M-HBs), and Small-HBs (S-HBs), known to contribute to HBV-driven pro-oncogenic properties. Here, we examined the kinetics of HBs-isoforms in virologically-suppressed patients who developed or did not develop hepatocellular carcinoma (HCC). This study enrolled 30 chronically HBV-infected cirrhotic patients under fully-suppressive anti-HBV treatment. Among them, 13 patients developed HCC. Serum samples were collected at enrolment (T0) and at HCC diagnosis or at the last control for non-HCC patients (median (range) follow-up: 38 (12–48) months). Ad-hoc ELISAs were designed to quantify L-HBs, M-HBs and S-HBs (Beacle). At T0, median (IQR) levels of S-HBs, M-HBs and L-HBs were 3140 (457–6995), 220 (31–433) and 0.2 (0–1.7) ng/mL. No significant differences in the fraction of the three HBs-isoforms were noticed between patients who developed or did not develop HCC at T0. On treatment, S-HBs showed a >25% decline or remained stable in a similar proportion of HCC and non-HCC patients (58.3% of HCC- vs. 47.1% of non-HCC patients, *p* = 0.6; 25% of HCC vs. 29.4% of non-HCC, *p* = 0.8, respectively). Conversely, M-HBs showed a >25% increase in a higher proportion of HCC compared to non-HCC patients (50% vs. 11.8%, *p* = 0.02), in line with M-HBs pro-oncogenic role reported in in vitro studies. No difference in L-HBs kinetics was observed in HCC and non-HCC patients. In conclusion, an increase in M-HBs levels characterizes a significant fraction of HCC-patients while under prolonged HBV suppression and stable/reduced total-HBs. The role of M-HBs kinetics in identifying patients at higher HCC risk deserves further investigation.

## 1. Introduction

Hepatocellular carcinoma (HCC) is the third leading cause of cancer death worldwide [1]. The lifetime risk of developing HCC is 10- to 100-fold greater for chronic hepatitis B virus (HBV) carriers than non-infected individuals [2]. In contrast with Hepatitis C virus (HCV), a substantial number of HBV-infected patients develop HCC without signs of liver damage [2], highlighting the existence of intrinsic HBV-mediated pro-oncogenic mechanisms [3].

In the last two decades, anti-HBV treatment with nucleos(t)ide analogues (NUCs) has resulted in remarkable improvements in the survival of patients with chronic hepatitis B and has determined a reduced incidence of HBV-related HCC [4,5,6]. These critical goals have been achieved by the capacity of the most recent NUCs to maintain long-term viral suppression in >90% of treated patients and, in turn, to reduce hepatic inflammation and to prevent or reverse liver fibrosis [7,8,9].

Nonetheless, the risk of HCC development is not abolished in patients with persistently undetectable HBV DNA in the serum and is maximized in the presence of cirrhosis [10]. In Caucasian patients with cirrhosis, the cumulative incidence of HCC during entecavir (ETV) or tenofovir (TDF) therapy was 12.7% and 15.5%, respectively, at year five of follow-up [11]. To date, accurate virological biomarkers that can predict HCC risk under anti-HBV treatment are still missing, particularly in the setting of serum HBV-DNA undetectability. Current guidelines suggest performing semiannual ultrasound examination, ± alpha-fetoprotein, for the surveillance of the patients at risk [12]. Profiling the risk of HCC in individual patients might impact the need of surveillance, in terms of simplifying the protocols and reducing costs. For this purpose, a number of scores have been proposed, based on the combination of clinical and laboratory variables [13].

Recent evidence has found a good degree of correlation between the levels of HBsAg and HCC development [14,15]. Indeed, among treated patients with undetectable viral loads, low HBsAg levels (<1000 IU/mL) were associated with the lowest risk of HCC onset [16] whereas high levels were associated with the development of this cancer [14], suggesting the potential contribution of this serological marker as an HCC predictor.

The hepatitis B surface antigen (HBsAg) consists of three different proteins encoded within the same S open reading frame (ORF S) by the alternate usage of translational start codons—(i) large HBsAg (L-HBs), consisting of preS-1, preS-2 and S regions; (ii) middle HBsAg (M-HBs) consisting of pre-2 and S regions and a small HBsAg, containing only an S region [17,18]. Thus, all the three HBs proteins share the same C-terminal S region, that represent the targets of the commercially available serological assays routinely used for HBsAg quantification. Consequently, these commercial assays permit quantification of only the overall amount of the three HBs forms, while they cannot distinguish the different contribution of the three HBs isoforms [19].

Recent data have risen on the interest in the differential quantification of HBs forms as novel biomarkers for HBV infection staging and for predicting therapeutic outcome [19]. In particular, recently it has been demonstrated that the composition of the HBsAg significantly changes across the different stages of HBV infection, showing lower proportions of L-HBs and M-HBs in the HBV chronic infection quiescent stage compared to those observed during chronic active hepatitis B [20]. Additionally, a recent study has also demonstrated that a decrease in L-HBs and M-HBs proportions (prior to total HBsAg decay) during NUC or pegylated interferon alpha (PEG-IFN) treatment can represent an early marker of favorable therapeutic outcome, preceding HBsAg loss [21].

Nevertheless, no data are still available on the levels of HBs forms and their kinetics in the setting of patients developing HCC, under fully suppressive antiviral treatment. In this light, we assessed the levels of HBs forms in virologically suppressed patients with cirrhosis and compared their proportions in patients developing or not HCC, to explore their potential role as a novel virological marker of HCC development.

## 2. Materials and Methods

### 2.1. Study Population

The study included Caucasian cirrhotic patients with chronic HBV infection ± Hepatitis Delta Virus (HDV) coinfection, on persistent HBV DNA suppression while treating with potent antiviral therapy. Excluded were patients with history of HCC or with alcohol use and human immunodeficiency virus (HIV) or HCV coinfection, furthermore, we excluded from the study the patients who developed HCC within six months after enrolment. The diagnosis of cirrhosis had been assessed on the basis of a previous liver biopsy in 11 patients (36.6%) or by the presence of clinical, laboratory and imaging signs of cirrhosis in the remaining cases. During the subsequent follow-up, clinical and laboratory evaluation and abdomen ultrasound examination were performed every six months or when required for clinical reasons. The clinical endpoint was the development of HCC; the diagnosis of HCC was confirmed by Magnetic Resonance Imaging (MRI) or Computed Tomography (CT), according to the European association for the Study of the Liver (EASL) recommendations [22].

For the purposes of the present study, two plasma samples under virological suppression were retrieved for each patient, at enrolment (T0) and at HCC diagnosis or at the last control before ending the follow-up for patients not developing HCC (T1). T0 is the time-point corresponding to the achievement of virological suppression (serum HBV-DNA < 20 IU/mL). All plasma samples had been stored at −40 °C.

### 2.2. Standard Laboratory Tests for HBV Infection and Liver Functionality

Serum HBV-DNA was quantified using the COBAS AmpliPrep-COBAS TaqMan HBV test (Roche Diagnostics, Basel, CH, Switzerland), with a lower limit of detection of 20 IU/mL and HBsAg was quantified using the Elecsys^®^HBsAgII assay (Roche Diagnostics, Basel, CH, Switzerland), with a lower limit of detection of 0.05 IU/mL. HDV infection was diagnosed by the presence of anti-HDV antibodies and HDV RNA in plasma, as previously described [23]. Liver function tests were performed by standard commercial methods.

### 2.3. Quantification of the Different Forms of HBsAg

The levels of the three different forms of HBsAg (Large, Middle and Small HBs) were quantified by using three different ad-hoc designed ELISA assays, developed in collaboration with Beacle Incorporation (Kyoto, Japan). These assays have been demonstrated to have high sensitivity (detection limit for each protein is 0.1 ng/mL) and a high specificity, taking advantage of a sandwich system using two types of antibodies, anti-PreS1, anti-PreS2 and anti-S, respectively.

Firstly, total serum HBsAg was quantified by using the kit HBs S Antigen Quantitative ELISA Kit, Rapid-II (defined in the formula below as S assay) (Beacle Inc., Kyoto, Japan), by targeting the S region, common to all the HBs forms. Subsequently, the kit HBs Pre-S2 Antigen Quantitative ELISA Kit, Rapid (Beacle Inc., Japan) was used to quantify the L- and M-HBs, targeting the Pre-S2 region (defined as Pre-S2 assay). Lastly, the L-HBs was quantified by the kit HBs Pre-S1 Antigen Quantitative ELISA Kit, Rapid-II (Beacle inc., Japan), targeting the Pre-S1 region (defined as Pre-S1 assay). For all assays, the experimental procedure was carried out according to manufacturer’s instructions.

The following formulas were applied in order to obtain the levels of:

S-HBs = (Quantification by S assay) − (Quantification by Pre-S2 assay + Quantification by Pre-S1 assay)

M-HBs = (Quantification by Pre-S2 assay) − (Quantification by Pre-S1 assay)

For each patient, total HBs, L-HBs, M-HBs and S-HBs levels were quantified in two samples, collected at the previously mentioned timepoints (T0 and T1) and each sample was processed at least in duplicate.

Total-HBs quantification by Beacle and Cobas assays showed high concordance according to the Spearman Test (Rho = 0.82, *p* < 0.001).

### 2.4. Ethical Considerations

The research was conducted on plasma samples obtained for clinically routine reasons and then stored, for which the informed consent had been obtained; all data were previously anonymized, according to the requirements by Italian Data Protection Code (leg. decree 196/2003). The study was notified to the local ethical committee, since under Italian law, biomedical research is subjected to formal approval by ethic committees only for interventional studies (art. 6 and art. 9, leg. decree 211/2003).

### 2.5. Statistical Analysis

For each HBs isoform, the receiver operating characteristic analysis was used to identify the variations associated with HCC onset. This analysis identified an increase >25% in M-HBs as the best and only cut-off capable to predict the onset of HCC. Conversely, no cut-offs were identified for S- and L-HBs. A Mann–Whitney test for continuous variables and Chi-Squared test for discrete variables were applied to define statistically significant differences. Statistical analysis was performed by using SPSS software (v19.0; SPSS Inc., Chicago, IL, USA).

## 3. Results

### 3.1. Study Population

Thirty patients were enrolled (median age 56.5 years, range 35–74)—11 were HBV mono-infected and 19 HDV coinfected. All had an undetectable HBV DNA in plasma and were under antiviral treatment from a median of 34 months (range 8–48); oesophageal varices were present in 14 (46.7%). After enrolment, they were followed up for a median of 38 months (range 12–48) (Table 1). Patients with HDV coinfection were younger than patients with HBV mono-infection (median (range) 58 years (39–73) vs. 66 (54–76); *p* < 0.05). None of the patients had an end stage liver disease; the MELD score ranged from 6 to 9.

During the follow-up, 13 patients developed HCC from 8 to 46 months after enrolment, namely seven out of 19 HDV-coinfected (37%) and six out of 11 HBV-mono-infected (54%). Patients with HCC were older than patients not developing HCC (66 (54–76) vs. 58 (39–75)) and had a more advanced cirrhosis as shown by the Child–Pugh score and the presence of oesophageal varices (all *p* < 0.05) (Table 2).

### 3.2. Quantification of HBs Forms at the First Time-Point (T0)

**Figure 1:**
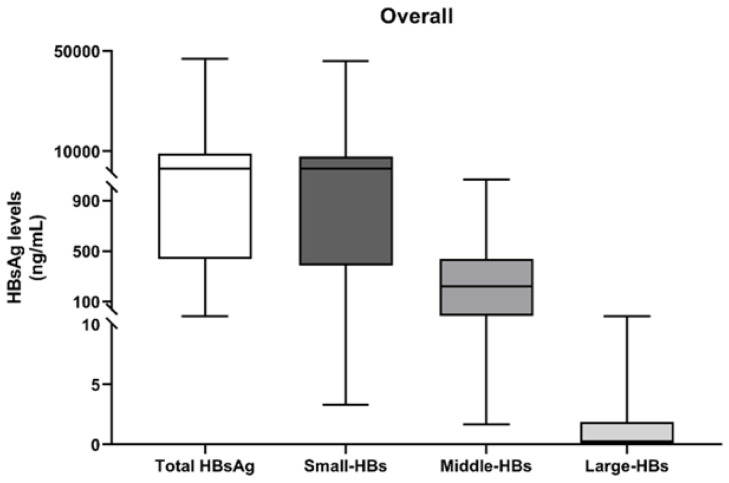
Levels at T0 of total-HBs, Small HBs S-HBs, Middle HBs (M-HBs) and Large HBs (L-HBs). The box plots report the distribution of values (ng/mL) for Total-HBs, S-HBs, M-HBs and L-HBs observed at the first analysed time-point (T0) for the overall study population (N = 30).

At T0, the median (IQR) levels of total-HBs in the overall population (N = 30) were 3188 (586–8161) ng/mL. By analysing the different HBs isoforms, the median (IQR) levels of S-HBs, M-HBs and L-HBs levels were 3140 (457–6995), 220 (31–433) and 0.2 (0–1.7) ng/mL, respectively (Figure 1).

By defining the contribution of each HBs isoform to the total-HBs in the overall population at T0, the median (IQR) ratios were 93.3% (84.5–95.7%) for S-HBs, 6.7% (4.3–15.5%) for M-HBs and only 0.01% (0–0.1%) for L-HBs isoform (Table 3), supporting that, in the setting of aviremic patients, total-HBs is mostly composed of S-HBs, with a not negligible amount of M-HBs and a very limited contribution of L-HBs.

At T0, no statistically significant differences emerged between patients developing HCC in the ratio of S-HBs (Median (IQR) ratios—90.0% (87.8–97.1%) in HCC vs. 93.5% (83.4–95.3%) in non-HCC group), of M-HBs (7.5% (2.9–12.1%) vs. 6.4% (4.6–16.5%)) and of L-HBs (0.01% (0–0.09%) vs. 0.01% (0–0.1%)) (Table 3).

Superimposable results were obtained when the analysis was focused on HDV-coinfected patients (Table S1).

### 3.3. On Treatment Kinetics of HBs Isoforms in HCC and Non-HCC Patients

A next step of this study was to investigate the treatment kinetics of HBs isoforms and their association with HCC onset. In particular, a receiver operating characteristic analysis showed that an M-HBs increase >25% was the best and the only cut-off positively associated with HCC onset, with a positive predictive value (PPV) and negative predictive value (NPV) of 75% and 71.4%, as well as with a diagnostic accuracy of 72.4%. Conversely, no specific cut-offs were identified for the other HBs isoforms.

In particular, an increase >25% of M-HBs levels was observed in a significantly higher proportion of HCC patients with respect to non-HCC patients (50% (6/12) vs. 11.8% (2/17), *p* = 0.023) (Table 4). In HCC patients, this increase ranged from 26% to 181%.

Of note, for three patients developing HCC, a serum sample was available collected at the last control before the first diagnosis of HCC (4–6 months before). In two of them an increase in M-HBs > 25% as compared to the basal value was detected.

Conversely, non-HCC patients were mainly characterized by stable levels of M-HBs during NUC treatment (64.7% (11/17) of non-HCC patients vs. 25% (3/12) of HCC, *p* = 0.035) (Table 4).

Lastly, an increase >25% in S-HBs and L-HBs was observed in a lower proportion of patients with no difference between HCC and non-HCC groups (16.7% (2/12) vs. 23.5% (4/17), *p* = 0.653 for S-HBs; 16.7% (2/12) vs. 29.4% (5/17), *p* = 0.430 for L-HBs) (Table 4).

A decline >25% in S-HBs (with respect to T0) was observed in a similar proportion of HCC and non-HCC patients (58.3% (7/12) of HCC vs. 47.1% (8/17) of non-HCC patients, *p* = 0.710). Likewise, the proportion of HCC and non-HCC patients with stable levels of S-HBs did not differ between the two groups (25% (3/12) vs. 29.4% (5/17)), *p* = 0.793) (Table 4).

## 4. Discussion

This study provides insights in the fractions of the different HBsAg forms in virologically suppressed patients with cirrhosis, their variation during long-term NUC treatment and their potential role as prognostic markers of HCC. In particular, this study (although on a small sample size) was the first to show an association between a rise (>25%) in M-HBs, and the onset of HCC. Of note, this increase in M-HBs occurred in the setting of declining or stable levels of total-HBs, observed in the vast majority of HCC patients. Overall, these data suggest a value of M-HBs (more than total-HBs) as a novel virological marker, contributing to predict HCC onset, in the setting of aviremic treated patients. This concept is further supported by analyzing an additional serum sample collected 4–6 months before HCC diagnosis, available for three patients, of whom two experienced an increase in M-HBs. Further studies on larger sample size are necessary to better clarify this point.

Conversely, our study also showed no different kinetics of L-HBs and S-HBs between the HCC and non-HCC group, suggesting that these two other HBs isoforms could be less informative in discriminating patients at higher risk to develop HCC.

The variations in M-HBs relative concentrations were found both in HBV-mono-infected and in HDV-coinfected patients who developed HCC. There is still uncertainty about the mechanisms involved in liver damage and cancer induced by HDV; an imbalance in gene activation in tumoral tissue, activation of oxidative and DNA methylation processes and HBV genome deletions have been proposed to occur [24,25,26]. Our data underscore the role of the M-HBs protein in this setting.

Overall, the results are in keeping with previous studies highlighting the involvement of M-HBs in hepatocarcinogenesis [27,28,29]. In particular, a previous study has shown that M-HBs can up-regulate the hTERT promoter, thus increasing the production of telomerase and in turn promoting cell proliferation [27]. Another study has shown that M-HBs can enhance the expression of forkhead box protein 3 (FOXP3) in a dose-dependent manner [29]. An increased FOXP3 production is known to play an important role in the neoplastic transformation of the hepatocytes [30]. Overall, these findings support the role of M-HBs as a potential viral oncoprotein and can explain the correlation between increased levels of M-HBs and HCC onset observed in our study. Further studies on larger sample size are necessary in order to confirm this intriguing issue.

It sounds plausible that the increased M-HBs production can be caused by mutations in the pre-S2 promoter that can upregulate the expression level of this open reading frame. These mutations could have been generated before starting NUC or during treatment. It is known that NUC treatment cannot completely abolish the production of infectious viral particles giving origin to a residual viremia and posing the basis for viral genetic evolution [31].

Furthermore, integrated HBV-DNA can represent a source for the production not only of S-HBs but also of M-HBs [32]. In this light, we cannot exclude that the increased M-HBs levels may be related to the integration of a pre-S2 containing genomic region that could potentially act as an additional source of M-HBs (beyond the circular covalently closed DNA (cccDNA)). HBV-DNA integration is considered a key mechanistic step underlying HBV-mediated carcinogenesis, even in the absence of necroinflammation [33,34,35]. Indeed, HBV DNA integration can compromise cell genome stability and modify the expression of genes regulating cell cycle/proliferation, predisposing the hepatocytes to pre-neoplastic transformation [34]. In this light, the integration of a pre-S2 containing genomic region could represent a source of M-HBs and at the same time put the basis for clonal expansion of the hepatocytes.

Considering the overall population, S-HBs was the most abundant HBs isoform in virologically suppressed patients during NUC treatment. Indeed, this isoform represents around 90% of total HBsAg, followed by M-HBs and L-HBs. It is known that L-HBs is present primarily in virions and plays an essential role in HBV infectivity since it contains the receptor-binding domain [36,37,38]. In this light, the suppression of viral particle production by NUC can explain the very low levels of L-HBs detected in our study population. Conversely, S-HBs is predominantly detected in non-infectious subviral particles whose production outnumbers the virions and is limitedly affected by NUC [38], thus explaining the abundance of this isoform in virologically suppressed patients.

During long-term NUC treatment, the three HBs isoforms tended to remain stable or undergo a decrease over time. This result is in line with a previous study describing the kinetics of the three HBs isoforms during treatment with NUC or IFN-alpha and their correlation with the achievement of HBsAg loss [21]. Interestingly, this study has highlighted that a strong decline of M-HBs is predictive of HBsAg loss during treatment with NUC or IFN-alpha, supporting the role of this HBs isoform as an early predictor of treatment response [21]. 

We are aware that the present study has some limitations, first of all the low number of patients, who all had cirrhosis and a long history of liver disease, and the lack of systematic blood sampling over time to fully assess the kinetics of HBs isoforms. Nevertheless, it explored a new field, i.e., the potential of HBs isoform dosage in predicting the outcome of HBsAg-positive patients with cirrhosis under prolonged antiviral therapy; we feel that the results may justify a wider study. Indeed, the identification of virological markers in the setting of HBsAg-positive subjects with liver disease and suppressed HBV replication represents an unmet medical need, answering the issue “assess host genetic and viral markers to determine prognosis and optimise patients’ management” raised by European HBV Guidelines promoted by EASL [12].

## 5. Conclusions

This study was the first to examine the kinetics of HBs isoforms (S-, M- and L- HBs) in virologically-suppressed patients who developed or did not develop HCC. Notably, we found that an increase in M-HBs levels characterizes a significant fraction of HCC patients despite prolonged HBV suppression and stable/reduced levels of S- and L-HBs. In this light, M-HBs might represent a potential novel biomarker contributing to predict liver disease progression in HBsAg-positive patients under prolonged fully-suppressive antiviral therapy. Indeed, in this setting, the identification of virological markers prognosticating clinical outcome still represents an unmet medical need, raised also by EASL HBV guidelines. For this reason, the role of M-HBs kinetics in identifying patients at higher HCC risk deserves further investigation.

## Figures and Tables

**Table 1:** Characteristics of the study population.

Patients’ Characteristics	N = 30
Males	22 (73.3)
Median age (range), years	56.5 (35–74)
HBV/HDV coinfection	19 (63.3)
HBV monoinfection	11 (36.6)
Cirrhosis	30 (100)
*Child-Pugh score* A5	16 (53.3)
A6	14 (46.7)
Oesophageal varices,	14 (46.7)
*Antiviral Therapy*	
Entecavir	26 (86.6)
Tenofovir	3 (10)
Other	1 (3.3)
Median follow up after enrolment (months)	38 (12–48)

Data are N (%), unless otherwise indicated.

**Table 2:** Characteristics of hepatocellular carcinoma (HCC) and non-HCC patients.

Patients’ Characteristics	HCC Patients (N = 13)	Non-HCC Patients (N = 17)	*p* Value
Males	11 (84.6)	11 (64.7)	0.222
Median age (range), years	66 (54–76)	58 (39–75)	0.049
HBV/HDV coinfection	7 (53.8)	12 (70.5)	0.346
HBV monoinfection	6 (46.2)	5 (29.4)	0.346
Child-Pugh A5	3 (23.1)	13 (76.5)	0.004
Child-Pugh A6	10 (76.9)	4 (23.5)	
Oesophageal varices	10 (76.9)	4 (23.5)	0.004

**Table 3:** Ratios of the different HBs isoforms at T0 in overall population and stratified according to HCC onset.

Percentages of HBs Forms ^a^	Overall (N = 30)	HCC Patients (N = 13)	Non-HCC Patients (N = 17)	*p*-Value
% S-HBs, Median (IQR)	93.3 (84.5–95.7)	90.0 (87.8–97.1)	93.5 (83.4–95.3)	0.968
% M-HBs, Median (IQR)	6.7 (4.3–15.5)	7.5 (2.9–12.1)	6.4 (4.6–16.5)	0.968
% L-HBs, Median (IQR)	0.01 (0–0.1)	0.01 (0–0.09)	0.01 (0–0.1)	0.849

^a^ Percentages were calculated as the median (IQR) ratio of each HBs isoform with respect to the total HBs.

**Table 4:** On treatment kinetics in the different HBs isoforms in HCC and non-HCC patients.

Total-HBs	HCC (N = 12) ^a^	No HCC (N = 17)	*p*-Value
Reduction>25% ^b^	6 (50)	8 (47.1)	0.876
Stable level ^c^	4 (33.3)	5 (29.4)	0.822
Increase>25% ^d^	2 (16.7)	4 (23.5)	0.653
S-HBs			
Reduction>25% ^b^	7 (58.3)	8 (47.1)	0.710
Stable level ^c^	3 (25.0)	5 (29.4)	0.793
Increase>25% ^d^	2 (16.7)	4 (23.5)	0.653
M-HBs			
Reduction>25% ^b^	3 (25)	4 (23.5)	*0.927*
Stable level ^c^	3 (25)	11 (64.7)	0.035
Increase>25% ^d^	6 (50)	2 (11.8)	0.023
L-HBs			
Reduction>25% ^b^	3 (25)	3 (17.6)	0.630
Stable level ^c^	7 (58.3)	9 (53.0)	0.774
Increase>25% ^d^	2 (16.7)	5 (29.4)	0.430

For total-HBs and each isoform (L-, M-, S-) the variation of values was analyzed at T1 respect to T0. ^a^ The kinetics of HBs isoforms were analysed in 12/13 HCC patients, due to the failed quantification of HBs isoforms in 1 sample at HCC diagnosis. ^b^ ”Reduction” indicates a decrease in the levels of total HBs and its isoforms at T1 > 25% at T1 respect to T0. ^c^ “Stable” indicates the levels of total HBs and its isoforms at T1 that differed <25% with respect to T0. ^d^ “Increase” indicates a rise in the levels of total HBs and its isoforms at T1 > 25% with respect to those at T0.

## Data Availability

The data that support the findings of this study are available from the corresponding author upon reasonable request.

## Supplementary Materials

The following are available online at https://www.mdpi.com/article/10.3390/microorganisms9040752/s1, Table S1: Ratios of the different HBs forms at T0 in HDV-coinfected patients stratified according to HCC onset.
