# Supplementary material for: An Increase in the Levels of Middle Surface Antigen Characterizes Patients Developing HBV-Driven Liver Cancer Despite Prolonged Virological Suppression
Source: Microorganisms. 2021 Apr 2;9(4):752. doi: 10.3390/microorganisms9040752 (PMC8065957; doi:10.3390/microorganisms9040752)
Supplement: Supplementary file 1 [file microorganisms-09-00752-s001.pdf]

Table S1. Ratios of the different HBs forms at T0 in HDV-coinfected patients stratified according to HCC onset

Percentages of HBs forms[a]	Overall (N=18)	HCC patients (N=6)	no-HCC (N=12)	P-value
**% S-HBs, Median (IQR)**	90.0 (64.6-95.0)	88.8 (63.5-92.3)	93 (69.3-95.2)	0.6
**% M-HBs, Median (IQR)**	9.9 (5.0-35.0)	11.1 (7.6-36.3)	6.7 (4.7-30.4)	0.3
**% L-HBs, Median (IQR)**	0.04 (0.01-0.26)	0.1 (0.03-0.2)	0.03 (0.01-0.3)	0.4

[a] Percentages were calculated as the median (IQR) ratio of each HBs forms respect to the total HBs
